# A Comparative Study on the Electrical and Piezoresistive Sensing Characteristics of GFRP and CFRP Composites with Hybridized Incorporation of Carbon Nanotubes, Graphenes, Carbon Nanofibers, and Graphite Nanoplatelets

**DOI:** 10.3390/s21217291

**Published:** 2021-11-02

**Authors:** Manan Bhandari, Jianchao Wang, Daeik Jang, IlWoo Nam, Baofeng Huang

**Affiliations:** 1 College of Civil Engineering, Nanjing Tech University, 30 Puzhu Road(S), Nanjing 211800, China; L20186110030@njtech.edu.cn (M.B.); jc.wang@njtech.edu.cn (J.W.); 2 Department of Civil and Environmental Engineering, Korea Advanced Institute of Science and Technology, Daejeon 34141, Korea; svs2002@kaist.ac.kr; 3 School of Spatial Environment System Engineering, Handong Global University, Pohang 37554, Korea; 4 College of Civil Engineering, Shanghai Normal University, Shanghai 201418, China

**Keywords:** carbon nanomaterials, polymer matrix composites, electrical properties, piezoresistive characteristics

## Abstract

In this study, hybridized carbon nanomaterials (CNMs), such as carbon nanotubes (CNTs)–graphene, CNT–carbon nanofibers (CNFs), or CNT–graphite nanoplatelet (GNP) materials were embedded in glass-fiber-reinforced plastic (GFRP) or carbon-fiber-reinforced plastic (CFRP) composites to obtain electrical/piezoresistive sensing characteristics that surpass those of composites with only one type of CNM. In addition, to quantitatively assess their sensing characteristics, the materials were evaluated in terms of gauge factor, peak shift, and R-squared values. The electrical property results showed that the GFRP samples containing only CNTs or both CNTs and graphene exhibited higher electrical conductivity values than those of other composite samples. By evaluating piezoresistive sensing characteristics, the CNT–CNF GFRP composites showed the highest gauge factor values, followed by the CNT–graphene GFRP and CNT-only GFRP composites. These results are explained by the excluded volume theory. The peak shift and R-squared value results signified that the CNT–graphene GFRP composites exhibited the best sensing characteristics. Thus, the CNT–graphene GFRP composites would be the most feasible for use as FRP composite sensors.

## 1. Introduction

Infrastructure typically refers to the fundamental services and systems that serve a country, city, or other areas, including the roads, bridges, tunnels, water supply, sewers, electricity grids, and telecommunication services that provide the basic necessities for a society to function [1]. Transportation-related infrastructure comprises a sizable portion of overall infrastructure and deteriorates over time. This is a significant issue in both developed and rapidly growing countries [2]. Moreover, the deterioration of transportation infrastructure has accelerated worldwide due to the effects of chemical de-icing agents and overloaded vehicles [3]. Because infrastructure plays a significant role across nations and in society, infrastructure damage can cause enormous social and economic losses.

Due to deterioration, many infrastructure components, such as bridges, have been subjected to load restrictions or replaced before reaching their intended service life. Replacing infrastructure is expensive; therefore, one solution involves the implementation of structural health monitoring (SHM) systems that can continuously monitor critical components [4].

SHM refers to a series of tasks such as the installment of sensors, measurement of parameters required for the assessment of structural health conditions, and the collection and interpretation of data [4,5]. Conventional SHM sensors include strain gauges and accelerometers, while recently developed sensors (or examination equipment) include fiber-optic sensors, such as fiber Bragg grating (FBG) sensors, and radiographic equipment [6,7]. These sensors possess both advantages and disadvantages [6]. For example, strain gauges and accelerometers can precisely measure the strain or displacement in localized areas where they are applied, but they are limited to quasi-point measurements [6]. If the sensors are not located directly at the damage site, they may not record any observable data [6]. Fiber-optic sensors offer some advantages over traditional quasi-point sensors, as they can be embedded in structures and capture changes in strain and temperature along their length [6]. However, fiber-optic sensors are brittle and often require artificial defects in the host structure to facilitate bonding between the sensor and the structure [6]. Lastly, radiographic equipment provides comparatively clearer images to show the extent and location of damage; however, expensive equipment and technical expertise are required to operate the equipment [6].

Recently, a piezoresistive (electrical resistance changes) sensing scheme without the drawbacks of conventional sensors was proposed. This scheme has attracted attention from researchers [8,9,10,11,12,13,14]. This piezoresistive sensing scheme can be used to fabricate composite sensor materials, and these composite materials can be employed in host structures in the forms of plates or wrappings over large areas. This would then enable the composite materials to detect changes in stress and strain over large areas. Moreover, this piezoresistive sensing scheme does not require expensive equipment or artificial defects in the host structure.

In 2010s, researchers suggested a novel sensing system using piezoresistive sensors in fiber-reinforced plastic (FRP) composites, which possessed SHM and structural strengthening functions [15,16]. In this polymer-based composite sensor, an electrically conductive filler was incorporated in the polymer, due to its insulating characteristics. Among the various conductive fillers, carbon nanomaterials (CNMs), such as carbon nanotubes (CNTs) and graphene, have been used as principal materials in numerous studies because of their exceptional mechanical and electrical properties, as shown in Table 1 [17,18,19,20,21,22]. However, some studies have reported drawbacks, such as limited mechanical and electrical properties and low sensing performance when the composites were fabricated with a single type of carbon nanomaterial [23,24].

To eliminate some of these drawbacks, the hybridization of one-dimensional CNMs (CNTs and carbon nanofibers (CNFs)) and two-dimensional CNMs (graphene and graphite nanoplatelets (GNPs)) was performed [13,14,37,38,39]. However, several studies have reported synergistic effects, indicating that further enhancement cannot be accomplished using a single type of CNM under the same conditions. This was demonstrated in terms of mechanical, electrical, and piezoresistive characteristics via hybridized CNT–graphene networks [13,22]. However, few have attempted to harness the performance of hybridized networks for the development of piezoresistive FRP composite sensors.

In this study, we developed piezoresistive FRP composite sensors by harnessing the synergistic effects of hybridized carbon nanomaterials to surpass the electrical and piezoresistive characteristics of existing CNM-incorporated composites. We used the following methods to create the hybridized-carbon-nanomaterial-embedded FRP composite and to enhance its feasibility.

(1) Different combinations of hybridized carbon nanomaterials were dispersed in an epoxy resin and applied onto glass-fiber- or carbon-fiber-woven fabrics to form the CNM-incorporated carbon-fiber-reinforced plastic (CFRP) or glass-fiber-reinforced plastic (GFRP) composites.
(2) The electrical properties were assessed using the two-probe method, and the piezoresistive sensing characteristics were examined by applying repeated tensile loads and synchronously monitoring changes in electrical resistance/stress.
(3) The piezoresistive sensing characteristics were assessed in terms of gauge factor, peak shift, and R-squared values.

## 2. Materials and Methods

### 2.1. Materials

The physical parameters of the four different CNMs (CNT, CNF, graphene, and GNP) used in this work are shown in Table 1, obtained from research performed by Wang et al. in 2020 [22]. Proprietary multi-walled CNTs, CNFs, and graphene were obtained from Daoking Co. Ltd. (Beijing, China), and proprietary GNPs were obtained from Timenano Co. Ltd. (Chengdu, China). We also used an epoxy resin and hardener produced by Xiangfeng New Composite Co., Ltd. (Kunshan, China), and the epoxy consisted of a 3:1 mix ratio of epoxy resin (E-4676) to hardener (HC-3008-5). These epoxy resins are known for their mechanical strength after curing, high adhesion, transparency, and good wettability. The physical parameters of the epoxy resin used in this work are shown in Table 2. In addition, glass-fiber-woven and carbon-fiber-woven plain fabrics, as well as Teflon insulation tape and conductive silver paste were used. The glass fiber fabric was produced by Suihua Glass Fiber Co., Ltd. (Jiangxi, China), and possessed physical characteristics including weather resistance, non-adhesive, chemical resistance, high insulation, high strength, good durability, as well as acid and alkali resistance. The carbon fiber fabric was produced by Miaohan Construction & Technology Co., Ltd. (Shanghai, China), and the physical parameters of the glass fiber and carbon fiber fabrics used in this study are shown in Table 3.

### 2.2. Mix Proportions

In this study, pure epoxy resin GFRP (and pure epoxy CFRP) without additives, CNT-incorporated FRP (CNT-only FRP), and two types of hybridized-CNM-incorporated FRPs were prepared. Three types of CNMs, graphene, carbon nanofibers (CNFs), and graphite nanoplatelet (GNP) sheets, were mixed along with the CNTs to produce CNT–graphene FRP, CNT–CNF FRP, and CNT–GNP FRP composites, respectively. The weight proportion of total CNMs was varied from 1.5 to 3 wt.% in the FRP composites. For the hybridized-CNM-incorporated composites, the ratios of the two CNMs were 1:1 [14,40]. Table 4 shows the mix proportions of the composite materials.

To precisely determine a range of percolation threshold, more compositions involving different content ratios of CNMs can be suggested. However, attempting to determine the precise range of percolation threshold is beyond the scope of the present study. Accordingly, two different CNM content ratios were selected by referring to previous experimental works, and the electrical/piezoresistive sensing characteristics were examined for them.

### 2.3. Sample Preparation

**Figure 1:**
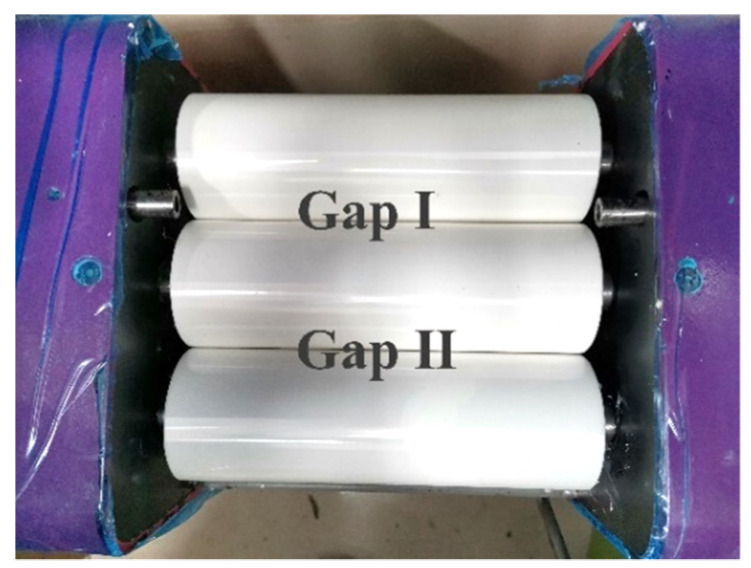
The three rollers and two gaps of a three-roll milling machine.

The CNMs, epoxy resin, and hardener were weighed according to the required amounts. After measuring, the materials were placed in a steel bowl and manually stirred for approximately two to three minutes. After preliminary mixing and stirring, to improve the dispersion of the CNMs in the resin, the CNM and epoxy resin mixture was passed through a three-roll milling machine (ZYE-50, Shenzhen Zhong Yi Technology Co. Ltd., Shenzhen, China), as shown in Figure 1.

**Figure 2:**
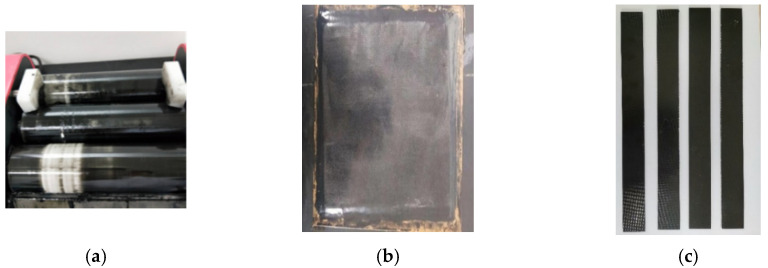
Fabrication procedures of the CNM-incorporated FRP composites (**a**–**c**). (**a**) Mixing by using a three-roll mill, (**b**) A multi-layered CNM-incorporated FRP composite made by hand lay-up, (**c**) Prepared FRP composite samples.

During dispersion, the epoxy resin and CNM mixture was squeezed through the small gaps between the three rollers via rotary extrusion. The CNM–epoxy mixture was first passed through a gap I (Figure 1), then passed again through gap II (Figure 1). The mixtures were milled and dispersed twice during a single milling process to complete one cycle. The gap distance between the rollers was controlled, with a specific range of 1–150 microns. Twelve repetitive cycles were conducted during the dispersion process (Figure 2a), and the gap distance was gradually reduced with each cycle. Table 2, from the research of Wang et al. in 2020 [22], shows the decrease in gap distance of gaps I and II as the number of milling cycles increased. After a total of twelve milling cycles, a good dispersion of the CNMs in the epoxy resin was confirmed by observing its dispersion through scanning electron microscopy (SEM), according to research conducted by Wang et al. in 2020 [22].

After dispersion, the CNM-incorporated epoxy resin was coated evenly on a 320× 230 mm aluminum plate and wrapped with Teflon insulating tape using a plastic spatula. After coating with the CNM-incorporated epoxy resin, the glass fabric was laid on top, and a consecutive coat of CNM-incorporated epoxy resin was applied on the surface of the glass fabric. This stacking process was repeated to create a total of five layers of glass fiber fabric for the GFRP and six layers of carbon fiber fabric for the CFRP composites. A layer of Teflon insulating tape was placed on the prepared composite surface to protect the outermost CNM-incorporated epoxy resin layer. A vacuum preservation machine (Shenzhen Airmate Technology Co., Ltd. (Shenzhen, China)) was then used to facilitate vacuum bagging (Figure 2b). After curing for 48 h, the GFRP composites were removed from the vacuum bag. The composites were then cut into rectangular strip plates 250 mm in length and 25 mm in width, according to the ASTM D 3039 standard (Figure 2c).

## 3. Test Methods

### 3.1. Electrical Resistance and Conductivity Test

**Figure 3:**
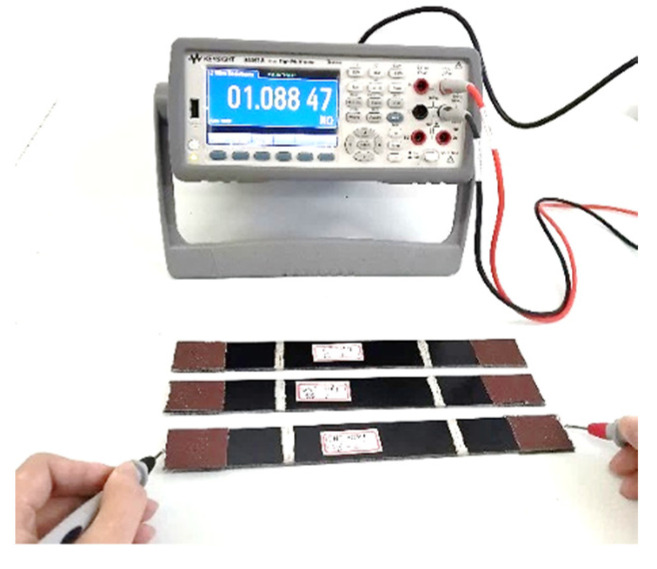
Electrical resistance measurements of the CNM-incorporated GFRP composites.

The electrical resistance of the CNM-incorporated FRP composites was measured using a two-probe method, and a digital multimeter (Keysight 34461A), shown in Figure 3, was used to measure the electrical resistance of the fabricated GFRP and CFRP composite samples. To reduce the contact resistance between the sample surfaces and the probe, a conductive silver paste was evenly coated on both test sample ends. To determine the representative electrical resistance of the samples, three replicated samples from each group were randomly selected. Then, the probe of the digital multimeter was placed in contact with the conductive silver paste layer and the resistance was measured, as shown in Figure 3. After the probe made contact for one second, the resistance data, as displayed by the digital multimeter, were recorded. Then, the resistance of the composites was calculated based on Ohm’s law. To determine DC conductivity, the following equation was used [22,41]:

(1) $$\sigma =\frac{1}{\rho }=\frac{L}{R\times A}$$

where *R* is the measured resistance value, *A* is the cross-sectional area of the silver paste layer in contact with the composite, and *L* is the interval of the electrodes. The silver paste was applied to both sides of the sample that were faced in opposite directions and used as electrodes.

### 3.2. Piezoresistive Sensing Performance Test

**Figure 4:**
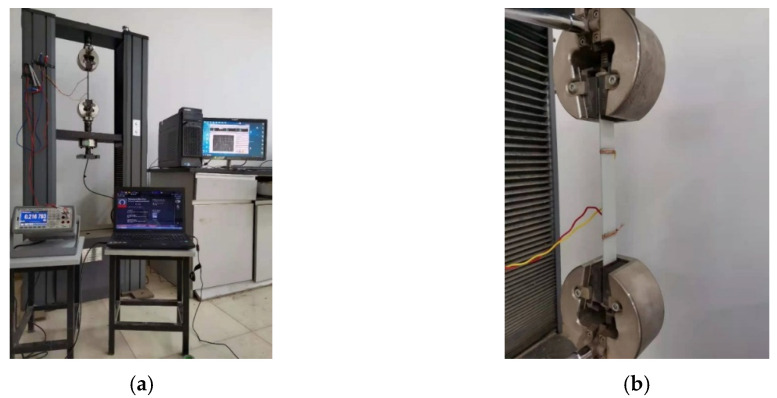
(**a**) The experimental set-up of the piezoresistive sensing test and (**b**) the CNM-incorporated FRP composites mounted in the UTM.

The experimental set-up and sample installment of the piezoresistive sensing test were as follows: To perform the piezoresistive sensing test, we used a universal testing machine (UTM) (Jinan Fangyuan Testing Instrument Co., Ltd., Jinan, China), and the applied loading data were automatically recorded by a computer, as shown in Figure 4a. To facilitate sample installment, sandpaper was attached to both sides of the sample. The sandpaper improved the contact surface roughness between the composite sample and the UTM, which increased the accuracy of the recorded data. Sandpaper also prevented direct contact between the metal fixtures of the UTM and the FRP composites. A layer of conductive silver paste was then uniformly coated 60 mm from both ends of the test sample and with a width of approximately 5 mm, as shown in Figure 4b. A copper wire was then wrapped around the surface of the two conductive silver coatings, and the two digital multimeter electrodes were connected externally. After mounting the test sample on the UTM, tensile loading was applied to the test sample at a rate of 1 mm/min and unloaded when the maximum tensile stress reached approximately 15 MPa. The loading and unloading cycles continued up to five times, and the electrical resistance and applied loading data were collectively recorded by the computer.

## 4. Results

### 4.1. Electrical Properties

**Figure 5:**
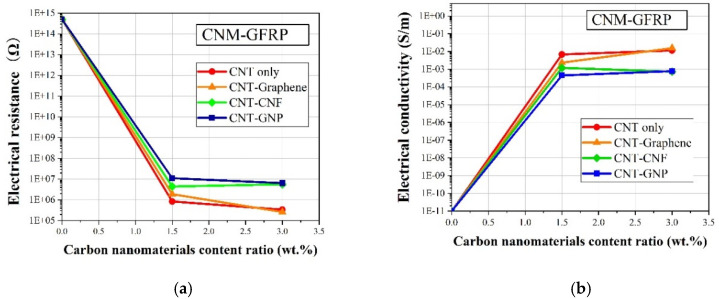
(**a**) The electrical resistance and (**b**) electrical conductivity of the CNM-incorporated GFRP composites.

Figure 5a shows the relationship between the electrical resistance of CNM-incorporated GFRP samples and the quantity of incorporated CNMs. The pure GFRP sample, without incorporated CNMs, exhibited an electrical resistivity greater than 100 GΩ∙m, as it was composed of insulating materials, namely epoxy resin and glass fiber [42]. Electrical conductivity networks formed in the GFRP, as the CNMs were incorporated in the GFRP, which reduced the electrical resistance of the GFRP samples. As shown in Figure 5a, the electrical resistance decreased dramatically when the incorporated CNM quantity was equal to or greater than 1.5 wt.%, regardless of CNM type. This result indicated that the incorporated CNMs in the insulating materials reduced their electrical resistance, changing their intrinsic properties from those of insulators to those of conductors. In addition, it was found that GFRP samples with just CNTs or both CNTs and graphene showed much higher electrical conductivity than the other samples that included GNPs or CNFs (Figure 5b). This was attributed to the electrical conductivity of the CNMs, indicating that the conductivity of CNT and graphene was higher than GNPs or CNFs. This result was in close agreement with results from previous studies [22]. Wang et al. (2020) investigated the effects of CNM type on the electrical conductivity of epoxy-based composites, and demonstrated that composites with CNT or graphene showed greater electrical conductivity than composites with CNFs or GNPs [22]. In addition, the percolation threshold phenomenon was observed in Figure 5a, indicating a dramatic reduction in electrical resistance as the incorporated CNM quantity increased [43]. As shown in Figure 5b, the percolation threshold was present from 0 to 1.5 wt.% of incorporated CNMs. This indicated that a percolated electrical network formed with incorporated CNM quantities of 1.5 to 3.0 wt.%. Furthermore, the CNT–graphene GFRP sample showed the highest electrical conductivity value among the samples including the 3 wt.% CNM sample. This result was deduced from the utilization of 2D-based CNMs, which improved the formation of electrical networks, as investigated in the literature [44]. Wang et al. (2016) reported that incorporated graphene in the prepared sheets was aligned in the in-plane direction during fabrication [44]. The electrical conductivity improved when the carbon-based layer was aligned in the in-plane direction, which was in good agreement with the results shown in Figure 5b [44].

**Figure 6:**
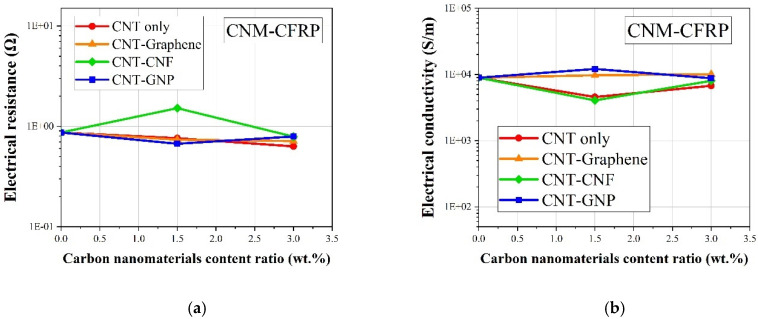
(**a**) The electrical resistance and (**b**) electrical conductivity of the CNM-incorporated CFRP composites.

The electrical resistance and conductivity values of the CNM-incorporated CFRP samples are shown in Figure 6a,b, respectively. The pure epoxy CFRP samples exhibited high electrical conductivity without the incorporation of CNMs, due to the high electrical conductivity of the CFRPs, which had a value of 8933 S/m as shown in Figure 6b. The electrical conductivity of the CNM-incorporated CFRP-based samples showed marginal variations within the ranges of a 0.5 order of the conductivity regardless of the incorporated CNM type, since the electrical conductivity value of the carbon fiber strands was about $3.8\times 10^{4}$ S/m, which was greater than that of the CNT and graphene used in this study, respectively [45].

### 4.2. Piezoresistive Responses of the Fabricated FRP Composites

**Figure 7:**
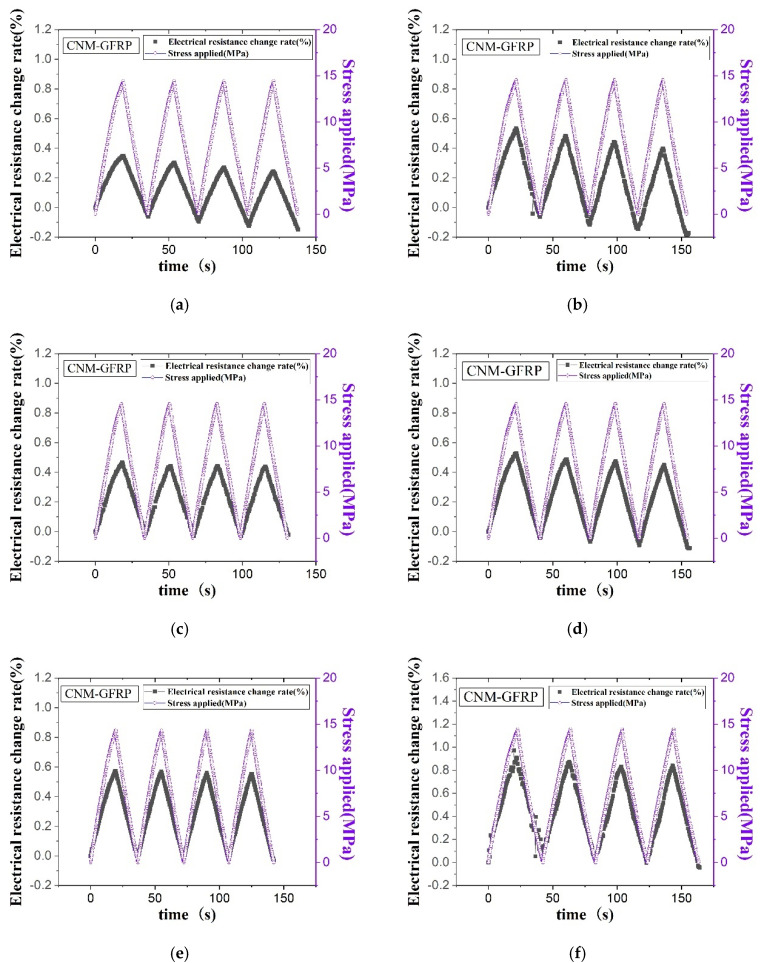
The electrical resistance change rate and applied stress of the (**a**) CNT-only GFRP 1.5% −2, (**b**) CNT-only GFRP 3% −1, (**c**) CNT–graphene GFRP 1.5% −3, (**d**) CNT–graphene GFRP 3% −1, (**e**) CNT–CNF GFRP 1.5% −1, and (**f**) CNT–CNF GFRP 3% −1 composites obtained through cyclic tensile loading tests.

Figure 7 shows the electrical resistance change rate and the applied stress of all GFRP composites containing just CNTs, both CNTs and graphene, and both CNTs and CNFs as a function of time. The results show an increase and decrease in electrical resistance as the tensile stress increased and decreased, respectively, which was typical of thermosetting-polymer-based sensing composites [46]. The piezoresistive characteristics of the composites containing CNMs, as derived from external loading, were due to the changes in contact resistance between the CNM particles and the deformation of the CNMs [13]. Because the latter had a smaller effect on the overall electrical resistance change compared to the former, changes in contact resistance were a major factor [13]. The change in contact resistance was further classified into changes in tunneling resistance and conductive pathways [13]. The change in tunneling resistance was caused by the tunneling effect, which refers to electrons hopping through spaces between the CNM particles without directly contacting the CNM particles [13]. The distance between the CNM particles where the tunneling effect occurred was a few nanometers. Once the conductive pathway weakened, changes in tunneling resistance became a major factor in changes in piezoresistive characteristics [13].

As shown in the piezoresistive sensing results, the baseline for each cycle of electrical resistance rate varied, regardless of CNM type. Studies on increasing the baseline with repeated cyclic tensile loading can be found in the literature, where it was correlated to the accumulation of damage in the CNM-incorporated composites. In contrast to the above phenomenon, in this study, the baseline tended to decrease with repeated cyclic tensile loading. This electrical resistance change rate trend has also been frequently reported in the literature. According to previous studies, the gauge length part of the sample was stretched in the longitudinal direction, and the width decreased in the transverse direction as the tensile loading was applied to the composites [19,47]. This action was responsible for the positive Poisson’s ratio of the composites [19,47]. Thus, transverse contraction led to the reconfiguration and/or reorientation of the three-dimensional CNM-based networks and created dense CNM-based networks, which decreased electrical resistance [19,47]. In the GFRPs composed of both CNTs and GNPs, the initial resistance value was too high; thus, the electrical resistance applied as external loadings exceeded the limitations that could be measurable via the two-probe method. Accordingly, the piezoresistive characteristics of the GFRP samples containing CNTs and GNPs will not be discussed.

**Figure 8:**
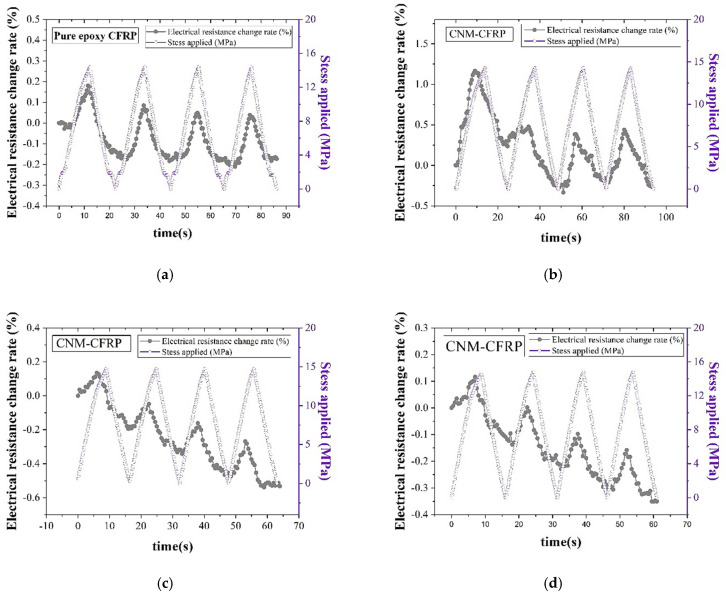

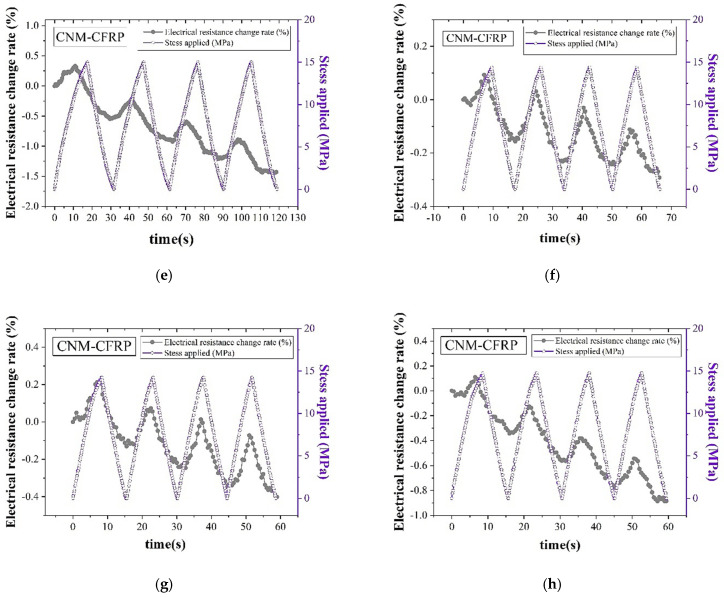
The electrical resistance change rate and applied stress of the (**a**) pure epoxy CFRP, (**b**) CNT-only CFRP 1.5%, (**c**) CNT–graphene CFRP 1.5%, (**d**) CNT–graphene CFRP 3%, (**e**) CNT–CNF CFRP 1.5%, (**f**) CNT–CNF CFRP 3%, (**g**) CNT–GNP CFRP 1.5%, and (**h**) CNT–GNP CFRP 3% composites obtained through the cyclic tensile loading tests.

Figure 8 displays that the sensing characteristics of the CFRP composites were less linear compared to the GFRP composites. It could have been expected that employing carbon fibers, which possess intrinsic electrical conductivity surpassing that of glass fibers, in FRP composites could contribute to enhancements in the piezoresistive sensing characteristics. However, several pieces of research found in the literature have shown that excessive inclusion of conductive fillers resulted in degradations of the sensing characteristics. Kostopoulos et al. (2009) investigated the influence of an increase in CNT content on piezoresistive sensing characteristics, and experimental results demonstrated that CNT incorporation exceeding 0.5% into CFRP composites caused adverse effects on piezoresistive sensing characteristics [48]. This can be explained with dense electrical networks. Owing to excessive incorporations of conductive fillers, dense, electrically conductive networks are initially formed in the composites, and notable changes in interconnections of the fillers were not attained even though external loadings were applied to the composites.

Among the CNM-incorporated CFRP composites, the CNT-only CFRP 3% composite was not included since it did not show an acceptable sensing response. Multiple CFRP 3% composites showed a decrease in electrical resistance under some parts of loading procedures rather than showing an increase in it. It can be speculated that the anomalous electrical resistance change responses can be ascribed to the low initial electrical resistance of the CNT-only CFRP 3% composite since it exhibited the lowest level among all CFRP composites, regardless of the CNM type and CNM content ratio. However, detailed investigations explaining the anomalous responses should be carried out, and they will be included in future works.

To quantitatively compare the sensing performance of the CRFP composites with each other, several factors were used for comparison.

### 4.3. Comparison of the Sensing Characteristics in Terms of the Average Maximum Electrical Resistance Change Rate and Gauge Factor

**Figure 9:**
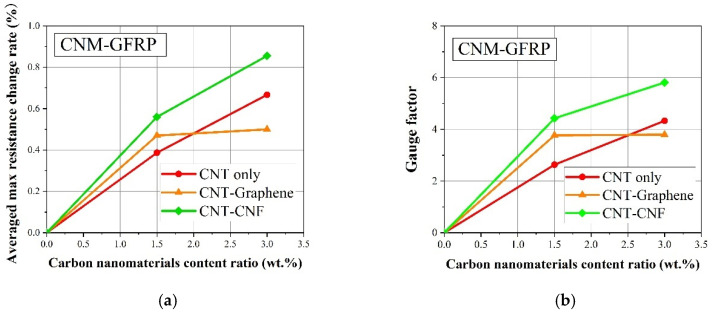
(**a**) Average maximum electrical resistance rate and (**b**) gauge factor of the CNM-incorporated GFRP composites.

Figure 9a shows the correlation between the average maximum electrical resistance rate of the CNM-incorporated GFRP composites and the CNM content ratio. The average maximum electrical resistance rate was determined by calculating the mean maximum electrical resistance rate from three replicated CNM-incorporated GFRP samples for each sample type.

Figure 9b shows the gauge factor of the CNM-incorporated GFRP composites as a function of the CNM content ratio. The gauge factor value was determined by a ratio of the maximum electrical resistance rate, *R,* to strain, *ε*, and refers to the electrical resistance change rate per unit strain [22]. Accordingly, the larger the gauge factor of a composite sample, the higher the electrical resistance that can be derived from the unit strain.

The average maximum electrical resistance rate and gauge factor were not determined for the control GFRP sample, without CNM, as no conductive networks formed in the material. The 1.5% and 3% CNMs formed conductive networks and yielded average maximum electrical resistance rate and gauge factor values. Specifically, the gauge factor obtained from the CNM-incorporated GFRP composites was comparable to the CNT-incorporated polymeric composites found in the literature [46]. The gauge factor results shown in Figure 9b varied within a range similar to that of the gauge factor shown in the authors’ previous study [22]. However, the difference in the trend of the gauge factor of the epoxy-based composites incorporating CNMs compared with that of the CNM-incorporated GFRP composites of the present study was exhibited. This was likely due to a change in microstructure stemming from the insertion of glass fiber fabrics; details of the affecting factors are subjects of further study.

**Figure 10:**
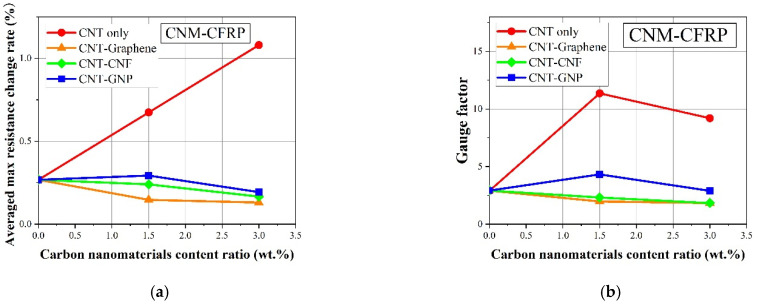
(**a**) Average maximum electrical resistance change rate and (**b**) gauge factor of the CNM-incorporated CFRP composites.

Figure 10a,b shows the average maximum electrical resistance change rate results and gauge factor of each CNM-incorporated CFRP composite type, with various CNM content ratios. The gauge factor of the pure epoxy CFRP fabricated without CNMs was 2.6. This was in contrast to the pure epoxy GFRP, which did not exhibit sensing characteristics. Compared to the gauge factor of pure epoxy CFRP, the CNT-only CFRP composite group only showed a prominent increase in the gauge factor with the addition of CNTs and reached a value of 8 at 3% CNT content.

The gauge factors decreased in all of the 3%-CNM-incorporated CFRP composites, which is explained by the sufficiently electrically conductive pathways formed by the carbon fibers and the relatively high CNM content ratio. Although breakage in the pathways due to external loading occurred, it did not significantly affect the overall conductive pathways as the proportion of broken pathways was reduced among the overall conductive pathways. Therefore, the gauge factor was reduced, though the CNM content ratio increased in the CFRP composites.

Among the different types of CNM-incorporated GFRP composites shown in Figure 9, the CNT–CNF GFRP group showed the highest values for the average maximum electrical resistance change rate and gauge factor, followed by the CNT–graphene GFRP and CNT–only GFRP groups. These different trends were dependent on the CNM type, which can be explained by the excluded volume theory, where the morphological effect of the CNM is considered [23,46,49].

Excluded volume refers to the filler-free volume adjacent to the CNMs, and where the center cannot penetrate in the composites with percolated networks of randomly oriented CNMs [23,46,49]. When rod- or disk-shaped fillers are packed in a limited three-dimensional space, they will pack more loosely than fillers with a spherical shape due to their geometrical characteristics [23,46,49]. Thus, the three-dimensional space with the loose packing will result in a higher degree of excluded volume. The excluded volume can be assessed by the following equations when rod-shaped or disk-shaped fillers are incorporated in a limited three-dimensional space [23,46], according to:

(2) $$Excluded\;volume\{\;\begin{array}{c} V_{Ex}^{r}=\left(\frac{\pi }{2}\right)L_{r}^{2}d_{r}+2\pi d_{r}^{2}L_{r}+\left(\frac{4}{3}\right)\pi d_{r}^{3}\\ V_{Ex}^{d}=\left(\frac{\pi^{2}}{8}\right)d_{d}^{3} \end{array}$$

where *L* and *d* indicate the length and diameter, respectively, and subscripts *r* and *d* denote the rod and disk, respectively [23,46].

A CNM-percolated network with a large degree of excluded volume will likely cause disruptions in the CNM network, as it is loosely packed. CNM disruptions can also cause electrical resistance changes, resulting in an increase in gauge factor [23,46]. In a previous study, CNMs with identical material properties to the present work were used to prepare CNM-incorporated epoxy composites without carbon or glass fiber fabrics, and the gauge factor was determined and explained by the excluded volume of the CNMs [22]. The excluded volume was the largest in the GNP networks, followed by the CNF, graphene, and CNT networks [22], as shown in Table 5. The previous study demonstrated that the excluded volume order of the CNM was the same as the evaluated gauge factor [22]. This outcome was also used in this study. In the piezoresistive characteristic experiments, the CNT–GNP materials were not considered. However, the other three composite group types, CNT-only, CNT–CNF, and CNT–graphene GFRP composites, were considered. Among the three types, the excluded volume of CNFs was the largest, followed by graphene and CNTs. The gauge factor results of the CNM-incorporated GFRP composites confirmed that the calculated excluded volume was closely related to the gauge factor [22].

### 4.4. Comparison of Sensing Characteristics in Terms of Peak Shift

**Figure 11:**
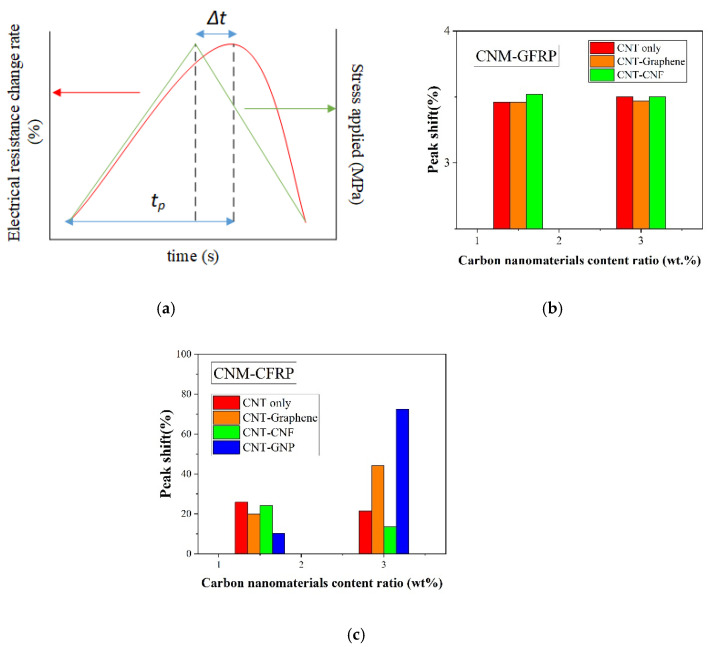
(**a**) Illustrative descriptions of the peak shift cf. [50], (**b**) calculated peak shift values of the CNM-incorporated GFRP composites, and (**c**) calculated peak shift values of the CNM-incorporated CFRP composites.

To quantitatively assess the effect of CNM and fiber fabric type on the piezoresistive sensing characteristics of the fabricated FRP composites, the sensing characteristics of the CNM-incorporated GFRP or CFRP composites were compared in terms of a time-domain factor, specifically the peak shift [50]. A peak shift consists of two physical parameters, where one is the time interval, ∆*t*, measured as the time between the peak of the electrical resistance change rate and the peak of applied stress. Another is the time, $t_{p}$, required to reach the point where the electrical resistance change rate is the highest from the start of the corresponding resistance change rate cycle [50]. The peak shift is a ratio of these two time-domain parameters and reflects the ability of the FRP sensing composites to convert the externally applied stress peak into a physical signal, which is the electrical resistance change rate in this study, without a time delay. Figure 11 shows ∆*t* and $t_{p}$ with illustrations, as well as the calculated peak shift values for the CNM-incorporated FRP composites. In addition, the formula of the peak shift is provided as follows [50]:

(3) $$Peak\;shift\;\left(\%\right)=\frac{\Delta t}{t_{p}}\times 100$$

Peak shift represents the electrical resistance changes in the composites with respect to applied load in real-time. The higher the peak shift of the composites, the slower the response time to convert the loading signals into the output of electrical signals. Conversely, the lower the peak shift of the composites, the faster the response time to convert the loading signal into the output of electrical signals.

The peak shift was obtained by averaging the peak shift of the three samples for each composite type. As shown in Figure 11b, all the peak shifts of the CNM-embedded GFRP samples were between 3.46 to 3.52%, indicating that they were not significantly affected by the CNM type or content ratio. The peak shift results demonstrated that the GFRP samples exhibited time-domain sensing performance comparable to the CNM-embedded polymeric composites described in the literature [22,24].

The peak shifts of the CFRP samples were calculated using the same methods, and the results are shown in Figure 11c. All CNM-incorporated CFRP composites had large peak shifts ranging from 10 to 73%, and even the smallest peak shift for the CNT–GNP 1.5% group still exceeded 10%, indicating that the composites did not exhibit good time-domain performance.

This phenomenon was attributed to the conductive carbon fiber fabric in the CFRP samples, which may have affected the piezoresistive sensing performance of the entirety of the CFRP samples. Therefore, additional studies are needed to further investigate the effect of carbon fiber fabric on the piezoresistive sensing performance of CNM-incorporated CFRP composites.

### 4.5. Comparison of the Sensing Characteristics in Terms of R-Squared

To quantitatively evaluate the effect of CNM and fiber fabric type on the piezoresistive sensing performance of CNM-incorporated FRP composites, sensing stability was assessed. Thus, the R-squared values were determined by using the cubic polynomial regression fitted from the applied loading and electrical resistance change rate values [22]. The R-squared results can indicate the degree of data dispersion between the applied loading and electrical resistance changes in each sample. If the applied loading and electrical resistance change data showed a small dispersion and a pronounced regularity, the R-squared would be close to 1.0. However, if the data dispersion became more scattered, the corresponding R-squared value would be smaller. This is explained by the definition of R-squared, which is also known as the coefficient of determination. According to the definition, the R-squared value becomes smaller as the differences between actual data and corresponding fitted data become larger.

**Figure 12:**
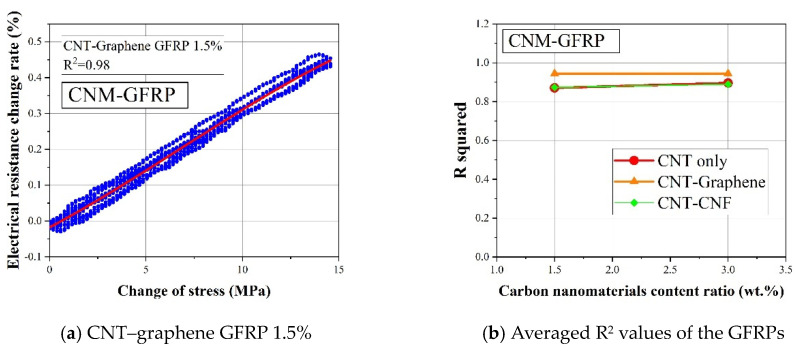
(**a**) Cubic polynomial regression fitted from the applied loading and electrical resistance change rates and (**b**) averaged R^2^ values of the CNM-incorporated GFRP composites.

The R-squared values of the CNM-incorporated GFRP samples are shown in Figure 12a,b. All GFRP samples had R-squared values equal to or higher than 0.8, except for one 1.5% CNT–CNF GFRP composite sample, which had an R-squared value of 0.75 [22]. This result indicated that the fabricated CNM-incorporated GFRP samples had stable and reliable electrical resistance change rates under external cyclic loading, as utilized in sensor applications.

In Figure 12b, it was observed that the data dispersion was relatively small as CNTs and graphene were simultaneously embedded in the GFRP composites, leading to R-squared values that were higher than the GFRP composites with other types or combinations of CNMs. Overall, it was observed that the CNM-embedded GFRP samples showed satisfactory sensing reliability with R-squared values of 0.8 or greater, and the CNT–graphene GFRP composites had the highest R-squared values among the GFRP-based composites [22]. Of note, the CNT–graphene GFRP composites also displayed excellent gauge factor and peak shift results, as described in the previous sections. The prominent sensing characteristics of the CNT–graphene conductive network were likely due to the relatively high specific surface areas of graphene and CNTs, which may have increased the contact probability of the two CNMs in the initial conductive networks, forming networks susceptible to externally applied loads [13].

**Figure 13:**
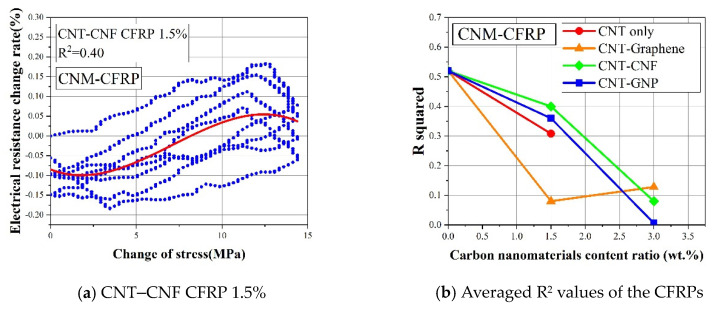
(**a**) The cubic polynomial regression fitted from the applied loadings and the electrical resistance change rates and (**b**) averaged R^2^ values of the CNM-incorporated CFRP composites.

Figure 13a,b shows the R-squared values of the CFRP-based composites. The R-squared values of the CNM-embedded CFRP samples were significantly lower than the CNM-embedded GFRP samples. Because the R-squared values of the CFRP-based samples were all less than 0.52, this indicated that the piezoresistive sensing reliability degraded, as the FRP composites were composed of carbon fiber fabric.

Figure 12b and Figure 13b show the averaged R-squared values of the GFRP- and CFRP-based composites, as determined from three replicated samples for each composite type. The figure also shows that the R-squared values increased once the CNMs were embedded in the GFRP-based composites (because the R-squared of pure epoxy GFRP was 0). However, the additional incorporation of CNMs, with content ratios of 1.5% to 3%, did not further improve the R-squared values, which indicated excessive embedment of CNMs. Thus, a 3% content ratio would no longer improve the sensing characteristics. In contrast to the GFRP-based composites, the R-squared values of the CFRP-based composites declined with the addition of CNMs, as shown in Figure 13b. Thus, considering the declining trend in the R-squared values of the CFRP-based composites, it was deduced that the excessive inclusion of conductive carbon materials in CFRP composites would be detrimental to their sensing characteristics.

When comparing the R-squared values of the CNM-embedded GFRP composites and the CNM-embedded CFRP composites, it was observed that the R-squared values of the CFRP-based composites were relatively lower than those of the GFRP-based composites. Kostopoulos et al. (2009) investigated the piezoresistive sensing characteristics of CNT-embedded CFRP composites and demonstrated that the noise in electrical resistance change rate was enlarged with CNT content ratios exceeding 0.5% [48]. This finding showed agreement with the declining trend in the R-squared values of the CFRP-based composites. In this regard, the effect of the excessive incorporation of CNMs and excessive inter-connections between CNMs and carbon fibers, which were speculated as principally affecting factors, on the degradation of sensing characteristics should be investigated further.

## 5. Conclusions

To overcome the drawbacks in the sensing performance of composites fabricated with a single type of CNM, hybridized CNMs such as CNT–graphene, CNT–CNF, or CNT–GNP materials were incorporated into epoxy resin and used to fabricate GFRP or CFRP composites. The fabricated CNM-incorporated FRP composites were evaluated in terms of their electrical properties and piezoresistive sensing characteristics. In particular, their sensing characteristics were assessed on the basis of parameters such as gauge factor, peak shift, and R-squared values. The experimental results can be summarized as follows:

(1) In the electrical property results, it was found that the GFRP samples with just CNTs or both CNTs and graphene showed much higher electrical conductivities than the other composite samples, and the percolation threshold was in the range of 0 to 1.5 wt.% of incorporated CNMs. Furthermore, the CFRP samples exhibited high electrical conductivity values, such as 8933 S/m, even without CNMs and marginal variations in CNM addition.
(2) After evaluating the piezoresistive sensing characteristics, it was found that the CNT–CNF GFRP composites exhibited the highest average maximum electrical resistance change rate and gauge factor values, followed by the CNT–graphene and CNT-only GFRP composites. These results were explained by the excluded volume theory, which yielded a higher excluded volume in the order of CNFs, graphene, and CNTs.
(3) All 3%-CNM-incorporated CFRP composites showed deterioration in terms of gauge factor, and this was ascribed to the adequately electrically conductive pathways formed by the carbon fibers and the relatively high CNM content ratio.
(4) All peak shifts of the CNM-embedded GFRP samples were in the range of 3.46 to 3.52%, signifying that the electrical resistance change rates of the composites were correlated to the applied loads. However, all CNM-incorporated CFRP composites had relatively large peak shifts, ranging from 10 to 73%.
(5) The fabricated CNM-incorporated GFRP samples showed more stable and reliable electrical resistance change rates, which accounted for their higher R-squared values, compared to the fabricated CNM-incorporated CFRP samples. Furthermore, the CNT–graphene GFRP composites exhibited the best R-squared value. Because the CNT–graphene GFRP composites showed better peak shift and gauge factor performance, these composites were the most feasible for use as FRP composite sensors.
(6) Although a synergistic effect was unclear in the electrical conductivity results, synergistic effects were pronounced in the CNT–CNF GFRP composites and CNT–graphene GFRP composites investigated in terms of the gauge factor and the R-squared value, respectively.

## Figures and Tables

**Table 1:** Comparisons of CNT and graphene materials in terms of their mechanical/electrical properties and advantageous aspects of piezoresistive characteristics.

Property	Carbon Nanotube	Graphene
Young’s modulus (TPa)	~1.25 (SWNT) [25] ~0.27–0.95 (MWNT) [26]	1 [27]
Tensile strength (Gpa)	~13–52 (SWNT) [28] ~11–63 (MWNT) [26]	130 [27]
Electrical conductivity (S·cm^−1^)	~0.17–2 × 10^5^ [29]	~10^6^ [30]
Thermal conductivity (W·m^−1^·K^–1^)	6600 (SWNT) [31] 3000 (MWNT) [32]	~3000–5000 [33]
Density (g/cm^3^)	1.33 [34]	2.2 [35]
Advantage in piezoresistivity	Tunneling effect (electron transfer without tube/tube contact) [10]	Relatively larger surface area in 2D, leading to an increase in contact probability [36]

**Table 2:** Physical properties of the epoxy resin.

Property	Epoxy Resin
Color	Colorless and transparent
Shrinkage rate (%)	<1
Elongation (%)	≈1.2

**Table 3:** Physical properties of the glass fiber fabric and carbon fiber fabric.

Type of Fabric	Glass Fiber Plain Fabric	Carbon Fiber Plain Fabric
Grade (g)	200, first level	200, first level
Thickness (mm)	≈0.12	≈0.11
Elongation at break (%)	≈3	≈3

**Table 4:** Composite materials and their mix proportions.

GFRP/CFRP Type	CNMs Weight (g)	Base Resin (g)	Hard- Ener (g)	Fiber Vol.% *
Pure epoxy GFRP/CFRP	0	150	50	29.3%/50.8%
CNT-only GFRP/CFRP 1.5%	3.05	150	50	32.4%/22.8%
CNT-only GFRP/CFRP 3%	6.09	150	50	23.1%/28.1%
CNT–graphene GFRP/CFRP 1.5%	1.525/1.525	150	50	26.7%/47.1%
CNT–graphene GFRP/CFRP 3%	3.045/3.045	150	50	23.1%/41.3%
CNT–CNF GFRP/CFRP 1.5%	1.525/1.525	150	50	30.0%/33.0%
CNT–CNF GFRP/CFRP 3%	3.045/3.045	150	50	24.0%/41.3%
CNT–GNP GFRP/CFRP 1.5%	1.525/1.525	150	50	29.3%/52.8%
CNT–GNP GFRP/CFRP 3%	3.045/3.045	150	50	30.0%/45.5%

* Glass fiber or carbon fiber volumetric content ratios were approximately estimated.

**Table 5:** Excluded volume determined by the geometrical features of the CNMs [22].

CNM Type	*d* (μm)	*L* (μm)	Excluded Volume (μm^3^)
CNT	0.008	20	5.0
Graphene	1.75		6.6
CNF	0.17	20	110.4
GNP	9		898.5
